# Factor H C-Terminal Domains Are Critical for Regulation of Platelet/Granulocyte Aggregate Formation

**DOI:** 10.3389/fimmu.2017.01586

**Published:** 2017-11-23

**Authors:** Adam Z. Blatt, Gurpanna Saggu, Claudio Cortes, Andrew P. Herbert, David Kavanagh, Daniel Ricklin, John D. Lambris, Viviana P. Ferreira

**Affiliations:** ^1^Department of Medical Microbiology and Immunology, University of Toledo College of Medicine and Life Sciences, Toledo, OH, United States; ^2^Department of Biomedical Sciences, Oakland University William Beaumont School of Medicine, Rochester, MI, United States; ^3^The School of Chemistry, University of Edinburgh, Edinburgh, United Kingdom; ^4^The National Renal Complement Therapeutics Centre, Newcastle upon Tyne, United Kingdom; ^5^Institute of Cellular Medicine, Newcastle University, Newcastle upon Tyne, United Kingdom; ^6^Department of Pharmaceutical Sciences, University of Basel, Basel, Switzerland; ^7^Department of Pathology and Laboratory Medicine, Perelman School of Medicine, University of Pennsylvania, Philadelphia, PA, United States

**Keywords:** factor H, platelet, neutrophil, complement, atypical hemolytic uremic syndrome

## Abstract

Platelet/granulocyte aggregates (PGAs) increase thromboinflammation in the vasculature, and PGA formation is tightly controlled by the complement alternative pathway (AP) negative regulator, Factor H (FH). Mutations in FH are associated with the prothrombotic disease atypical hemolytic uremic syndrome (aHUS), yet it is unknown whether increased PGA formation contributes to the thrombosis seen in patients with aHUS. Here, flow cytometry assays were used to evaluate the effects of aHUS-related mutations on FH regulation of PGA formation and characterize the mechanism. Utilizing recombinant fragments of FH spanning the entire length of the protein, we mapped the regions of FH most critical for limiting AP activity on the surface of isolated human platelets and neutrophils, as well as the regions most critical for regulating PGA formation in human whole blood stimulated with thrombin receptor-activating peptide (TRAP). FH domains 19–20 were the most critical for limiting AP activity on platelets, neutrophils, and at the platelet/granulocyte interface. The role of FH in PGA formation was attributed to its ability to regulate AP-mediated C5a generation. AHUS-related mutations in domains 19–20 caused differential effects on control of PGA formation and AP activity on platelets and neutrophils. Our data indicate FH C-terminal domains are key for regulating PGA formation, thus increased FH protection may have a beneficial impact on diseases characterized by increased PGA formation, such as cardiovascular disease. Additionally, aHUS-related mutations in domains 19–20 have varying effects on control of TRAP-mediated PGA formation, suggesting that some, but not all, aHUS-related mutations may cause increased PGA formation that contributes to excessive thrombosis in patients with aHUS.

## Introduction

The complement alternative pathway (AP) is a key component of the innate immune system. Not only does it maintain a low level of spontaneous activity in the fluid phase but it also amplifies complement activity initiated by the classical and lectin pathways. C3b deposited on cell surfaces by any complement pathway acts as a locus for AP activation. Factor B binds C3b and is subsequently cleaved by Factor D to form the C3 convertase, C3bBb. C3bBb cleaves more C3 to C3b that can bind covalently to cells and recruit Factor B to form additional AP C3 convertases. C3b bound, or in close proximity, to C3bBb [C3bBb(C3b)*_n_*] switches the specificity of the enzymatic complex from C3 to C5, leading to terminal complement activation that generates the effector molecules C5a and the membrane attack complex (MAC). Due to its spontaneity and its ability to amplify all complement activity, the human host has developed many regulatory mechanisms to prevent excessive AP activation (1).

Factor H (FH) is the key regulator of the AP in the fluid phase and also functions to limit AP activity selectively on self-cell surfaces (2). It accelerates the decay of C3/C5 convertases, and acts as a cofactor for Factor I-mediated cleavage of C3b to inactive C3b (iC3b) to prevent further convertase formation (3, 4). Twenty homologous complement control protein domains comprise FH. The four most N-terminal domains (domains 1–4) contain all of the protein’s regulatory function, whereas the remaining domains help anchor the protein to cell surfaces for efficient regulation by domains 1–4 (2). At least two distinct C3b and polyanion binding sites have been described between domains 7–20 (5). While these binding sites have cell- and tissue-specific roles in the human host (6, 7), the C-terminal domains, 19–20, contain both C3b and polyanion binding sites and are key for FH interactions with erythrocytes (8, 9), platelets (10–13), endothelial cells (13–15) and neutrophils (16), among other ligands.

Mutations in domains 19 and 20 that impair their ability to bind to C3b and polyanions are associated with atypical hemolytic uremic syndrome (aHUS) (9, 17, 18), a disease characterized by renal failure, hemolytic anemia, and thrombocytopenia (19). Thrombocytopenia is a result of excessive platelet activation leading to increased thrombi formation. While platelet–platelet and platelet–endothelium interactions are key for thrombi formation, activated platelets can also bind granulocytes to form platelet/granulocyte aggregates (PGAs). Increased PGA levels enhance thromboinflammation in animal models of vascular disease and are found in patients suffering from a variety of inflammatory vascular conditions, including acute coronary syndromes, inflammatory bowel and lung disease, and diabetes (20–22), however, it is not known whether increased PGA formation contributes to aHUS pathophysiology.

Complement activity enhances PGA formation in human whole blood stimulated with thrombin receptor-activating peptide (TRAP) (23–25). Our laboratory has previously shown that the AP is key to the effects of complement on PGA formation and that a competitive inhibitor of FH C-terminal domains 19 and 20 (rH19–20), significantly increases AP activity and enhances PGA formation in TRAP-stimulated human whole blood (25). By limiting critical FH cell-surface interactions and maintaining fluid-phase AP regulation, rH19–20 simulates the pathophysiological mechanisms involved in aHUS (8), suggesting that increased PGA formation could enhance vascular pathology in patients suffering from the disease. Here, we sought to better characterize the interaction of FH at the platelet/granulocyte interface and to determine the effects that mutations in domains 19 and 20 have on control of PGA formation in human whole blood.

## Materials and Methods

### Buffers

Modified HEPES/Tyrode’s (HT) buffer (137 mM NaCl, 2.8 mM KCl, 1 mM MgCl_2_ 6H_2_O, 12 mM NaHCO_3_, 0.4 mM Na_2_HPO_4_, 10 mM HEPES, 0.35% BSA, 5.5 mM glucose; pH 7.4); PBS (10 mM NaH_2_PO_4_, 140 mM NaCl, pH 7.4); Tyrode’s buffer [136.9 mM NaCl, 2.7 mM KCl, 983.8 µM MgCl_2_ 6H_2_O, 3.2 mM Na_2_HPO_4_, 3.5 mM HEPES, 0.35% BSA, 5.5 mM dextrose, 2 mM CaCl_2_ (pH 7.4)]; Tyrode’s + EDTA (Tyrode’s buffer + 10 mM EDTA); Tyrode’s + heparin + PGE_1_ (Tyrode’s buffer + 1 µM PGE_1_ and 2 IU/ml heparin); and MgEGTA [0.1 M MgCl_2_ and 0.1 M EGTA (pH7.3)].

### Detection Antibodies

Murine monoclonal antibodies (all IgG1κ): antihuman CD42b-APC (BioLegend), antihuman CD45-PE (BioLegend), antihuman/mouse C3/C3b/iC3b-FITC and unlabeled (Cedarlane), antihuman CD11b-PerCP/Cy5.5 and -PE (BioLegend), antihuman CD62P-PE/Cy5 (BioLegend), and goat antimouse-AF488. Isotype controls: APC- and PerCP/Cy5.5-labeled (BioLegend), PE-labeled (BioLegend), and FITC-labeled (Cedarlane).

### Serum Sources

Properdin-depleted serum and C8-depleted serum were purchased from Complement Technologies.

### Production of Recombinant FH Fragments

RH19–20 was produced as previously described (8). Additional coding sequence for residues rH1–3, rH2–4, rH3–5, rH6–8, rH7–9, rH8–10, rH10–12, rH12–14, rH12–15, rH13–15, rH14–16, rH15–17, rH16–18, and rH17–19 were PCR amplified (Table 1) from a full-length FH cDNA template (26), accession number NM_000186. The SapI-digested PCR products were cloned into pPICZ-α vector (Invitrogen) containing a His-6 tag and modified to include SapI sites in its multiple cloning site, as previously described (27, 28). Following transformation into *Pichia pastoris* strain KM71H, protein expression was induced with methanol following manufacturer’s instructions, deglycosylated by incubation with endoglycosidase H (New England Biolabs, Ipswich, MA, USA), and the proteins were purified from the media by affinity chromatography using Ni-NTA Agarose (Qiagen).

**Table 1:** Primers used to amplify coding sequences of several fragments of Factor H.

Domains	Forward primer 5′ → 3 (ATATGCTCTTCATGC…)	Reverse primer 5′ → 3 (TATAGCTCTTCAACA…)
SCR 1–3	AATGAACTTCCTCCAAGAAGAAA	CTTTGGTTTCTCTTTACTCCAAAAAC
SCR 2–4	GGACATCCTGGAGATACTCC	TGAAGGCAACGGACGCCATC
SCR 3–5	TTACCAGTGACAGCACCAGA	TCTCGGAGCAGGTATCCAGC
SCR 6–8	GATTATCCAGACATTAAACATGGA	CGTGGGTTGAGCTGACCATC
SCR 7–9	TATTTTCCTTATTTGGAAAATGGATATAA	TATGGGTAAATCAGACCAACCA
SCR 8–10	TCCAAATCAAGTATAGATATTGAG	TATTGGGAGGTCAGGAGACA
SCR 10–12	GAACTTCCTAAAATAGATGTACACT	CTGGGGAAGTTGGGTCCAT
SCR 12–14	GGAGATATACCTGAACTTGAACAT	GAGTGGTATTGACTGCCATCT
SCR 12–15	GGAGATATACCTGAACTTGAACAT	CTGAGGTGGAGAACTCCATTTTC
SCR 13–15	GGAGATATACCTGAACTTGAACAT	CTGAGGTGGAGAACTCCATTTTC
SCR 14–16	CCACCTCCACCTCAGATTC	TGATGGAGGGTGAGACCATTT
SCR 15–17	TCACAACCACCTCAGATAGAAC	TGTTGGCCTTCCTGTCCATC
SCR 16–18	AAATCTCCACCTGAGATTTCTCAT	TTGAGGTGGTTCCGTCCAG
SCR 17–19	CTCAGTTTACCTAGCTTTGAAAATG	TTTTGGTGGTTCTGACCATTGTC

*Underlined are the nucleotide sequences containing the SAPI restriction site that are added in each primer*.

RH19–20 mutants were produced as previously described (9). Two additional mutants, W1183L and R1215Q, were produced as described in the cited reference (9). Concentrations for all proteins were determined (280 nm), in triplicate, using theoretical ${\text{E}}_{1\text{ cm}}^{1\%}$ values (e.g., 1.49 for W1183L and W1183R and 1.85 for the rest of the mutants) using the Expert Protein Analysis System (EXPASY; http://www.expasy.org/tools/protparam.html).

### Complement Inhibitors

Cp20 (Ac-I[CV-1MeW-QDW-Sar-AHRC]mI-NH_2_), a potent compstatin analog, was used to inhibit convertase-mediated C3 activation, and was produced by solid phase synthesis (29). PMX53 (30) was produced as described. SALO (31) was a generous donation from Dr. Jesus Valenzuela (National Institutes of Health). Eculizumab was purchased from Creative Biolabs, and OmCI (32) was a generous donation from Dr. Susan Lea (University of Oxford). Human IgG4 isotype control (BioLegend) was used as a control for Eculizumab.

### Purification of Properdin and Separation of Physiological Properdin Forms

Properdin was isolated from human plasma, as previously described (33). For some experiments, properdin dimers, trimers, and tetramers (P_2_, P_3_, and P_4_) were separated from non-physiological aggregates (P_n_), stored at 4°C, and used within 2 weeks of separation, as previously described (33, 34).

### *Ex Vivo* Whole Blood Assay for Detecting PGA Formation

Human whole blood was obtained *via* venipuncture from volunteer donors. The Institutional Review Board from the University of Toledo College of Medicine and Life Sciences approved the protocols, and written informed consent was obtained from all donors, in accordance with the Declaration of Helsinki. Blood was drawn into vacutainer tubes (Becton Dickinson) containing 50 µg/ml final concentration of the thrombin inhibitor lepirudin (Celgene). 20 µl of whole blood was gently mixed with modified HT buffer + 10 µM TRAP (Bachem) and complement modulators, for a final volume of 80 µl. All groups were set up in duplicate. Samples were incubated at 37°C for 15 min and the reaction was stopped by addition of 800 µl RBC lysation/fixation solution (BioLegend). Samples were fixed for 10 min at room temperature (RT) before being washed, stained with detection antibodies for 15 min at RT, then diluted with 800 µl RBC fixation/lysation solution. Finally, samples were spun at 200 g for 15 min at 4°C, then 500 µl supernatant was removed and the samples run on a FACSCalibur (Becton Dickinson) flow cytometer. 10,000 events were acquired from a gate encompassing granulocyte and monocyte populations. Using FlowJo software version 7.6 (Tree Star), granulocytes were gated based on CD45 and side scatter, and the percent of granulocytes positive for CD42b fluorescence, as well as the C3- and CD11b-associated geometric mean fluorescent intensities (GMFIs) on gated granulocytes, were determined.

### Detection of C5a

Whole blood assays were set up as described, but in triplicate. Following the 37°C incubation, one replicate was immediately placed on ice then spun at 300 g for 10 min at 4°C. The other two replicates were processed *via* flow cytometry, as described above. Supernatants were spun at 13,000 g for 5 min at 4°C, then immediately frozen at −80°C until use. Supernatants were diluted 1/10 and C5a levels determined *via* standard ELISA kit (Abcam) following manufacturer’s instructions.

### Platelet Isolation and Activation

Platelets were isolated from human whole blood as previously described (12). Briefly, blood from volunteer donors was drawn into acid citrate dextrose vacutainers *via* venipuncture then spun at 200 g for 15 min at RT without braking. Platelets were washed twice with acid citrate wash buffer at 440 g for 10 min at RT, then resuspended in Tyrode’s buffer. Platelets (1 × 10^8^/ml) were activated with thrombin (1 U/ml) for 30 min at 37°C and the reaction stopped by the addition of Tyrode’s + heparin + PGE_1_, as previously described (12).

### Isolation of Neutrophils

Neutrophils were isolated from EDTA-anticoagulated human whole blood *via* Polymorphprep gradient centrifugation, using the manufacturer’s protocol (12). Briefly, whole blood was layered over Polymorphprep in a 1:1 ratio, then spun at 500 g for 35 min (without brake) at RT. The neutrophil layer was obtained from the gradient and washed with an equal volume of half-strength HBSS, without Ca^+2^ or Mg^+2^ (Life Technologies), at 400 g for 10 min at RT. Contaminating erythrocytes were lysed by a 7-min incubation in RBC lysis buffer (BioLegend) at 37°C, pelleted at 400 g for 10 min at RT, then finally resuspended in HBSS + 0.2% BSA.

### Measurement of AP Activation on Platelets

For mapping experiments, non-activated or thrombin-activated platelets were preincubated with 25 µg/ml properdin oligomers (P_2_–P_4_) in a 1:2:1 ratio for 1 h at RT, as previously described (12). Platelets (2 × 10^7^/ml) were washed, then incubated with 18 µM rH fragments, 5 mM MgEGTA, and 60% properdin-depleted serum for 30 min at 37°C in a total reaction volume of 100 µl. The reaction was stopped by washing samples with cold Tyrode’s + EDTA. C3 fragment deposition on the platelet surface was assessed by incubation with unlabeled anti-C3/C3b/iC3b for 60 min at 4°C, followed by AF488-goat-antimouse for 45 min at 4°C. Platelets were then stained with anti-CD42b and anti-CD62P for 45 min at 4°C, before being fixed with 1% paraformaldehyde for 30 min at 4°C. Platelets were gated *via* FSC and CD42b-fluorescence and at least 10,000 events were acquired.

For rH19–20 mutant studies, the experimental set-up was identical with the following exceptions: (1) platelets were incubated with 1 µg/ml unfractionated properdin rather than properdin oligomers to induce complement activation on the thrombin-activated platelet surface. 1 µg/ml of unfractionated properdin activates complement to the same extent as 25 µg/ml P_2_–P_4_ on platelets (not shown) due to the presence of aggregated properdin in unfractionated preparations (12, 33); (2) thrombin-activated platelets were used at 1 × 10^7^/ml during the incubation with properdin-depleted serum; (3) thrombin-activated platelets incubated in the presence of wild-type (WT) rH19–20 and Cp20 were used as the negative control in lieu of non-activated platelets; and (4) C3 fragment deposition was assessed using a FITC-labeled anti-C3/C3b/iC3b monoclonal antibody, simultaneously with the anti-CD42b and anti-CD62P incubation.

### Measurement of AP Activation on Neutrophils

For both mapping and rH19–20 mutant studies, isolated neutrophils (2 × 10^6^/ml) were mixed with HBSS + 0.2% BSA, rH constructs and other complement modulators (Cp20 and FH) in the presence of C8-depleted serum (33%) and 2.5 mM MgEGTA or 10 mM EDTA (negative control) for 5 min at 37°C. Final concentrations of rH constructs and other reagents are indicated in figure legends. Reactions were stopped with the addition of cold HBSS + 0.2% BSA containing 10 mM EDTA. Cells were washed then stained with anti-C3/C3b/iC3b-FITC and anti-CD11b-PE for 15 min at 4°C before being run on the flow cytometer. Neutrophils were gated *via* SSC and CD11b-fluorescence and propidium iodide was used for live/dead cell discrimination. At least 5,000 events (live neutrophils) were acquired, and the C3-associated GMFI on live neutrophils was determined.

### Statistics

GraphPad Prism 6.0 was used to analyze data. One-way ANOVA with multiple comparison tests (indicated in figure legends) was used to determine statistical significance between groups. *p* values less than 0.05 were considered statistically significant.

## Results

### Domains 19 and 20 Are Most Critical for FH Regulation on Cells Involved in PGA Formation

Platelets (12) and neutrophils (35) both activate the AP on their surface. Isolated platelets and neutrophils, in the absence of serum, bind FH primarily *via* its C-terminus (10, 11, 16). FH binds to multiple ligands (gpIIb/IIIa and tsp-1) on isolated platelets (10, 36), however, it is unknown what regions contribute to FH function when the AP is activating. Although Saggu et al. (12) determined that the C-terminus of FH is important for its interaction with platelets during complement activation, no one has systematically tested whether other domains of FH contribute to binding and protecting the platelet surface. Also, it remains unknown how FH interacts with neutrophils to protect them in the presence of serum. Therefore, we mapped FH protection from complement on the surface of isolated platelets and neutrophils to determine the regions most critical for FH cell surface protection on cells involved in PGA formation.

**Figure 1:**
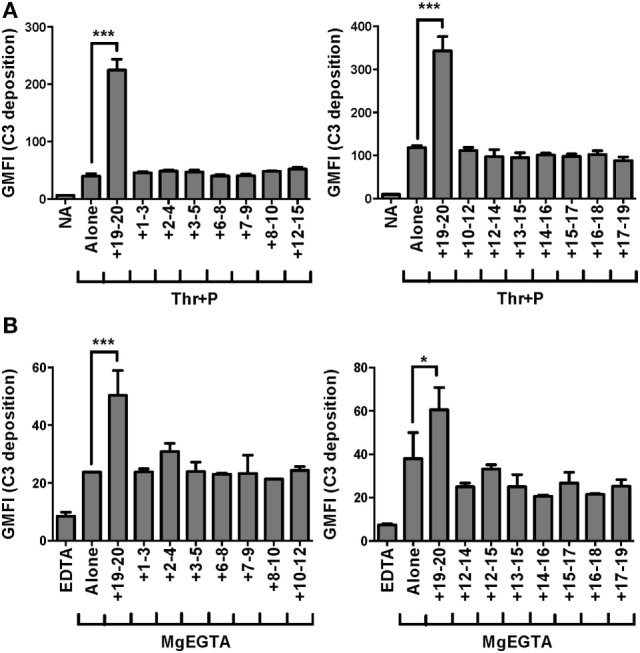
Factor H (FH) C-terminal domains are most critical for control of the alternative pathway on platelets and neutrophils. **(A)** Thrombin-activated platelets preincubated with 25 µg/ml P_2_–P_4_ (Thr + P; 2 × 10^7^/ml) were incubated without or with rH fragments (18 µM) in the presence of 5 mM MgEGTA and 60% properdin-depleted serum. Assuming 3 µM FH in serum, the molar ratio of rH:FH was ~10:1. Non-activated (NA) platelets were included as negative controls. C3 fragment deposition on platelets was determined as described in Section “Materials and Methods.” Results are representative of independent experiments done with platelets from different human volunteer donors that tested the entire rH fragment panel and are graphed as mean and SD of triplicate observations, *n* = 2. The data were analyzed by one-way ANOVA with Dunnett’s multiple comparison test against Thr + P. *p* < 0.001 (***). **(B)** Neutrophils (2 × 10^6^/ml) were incubated without or with rH fragments (20 µM) and 33% C8-depleted serum in the presence of 2.5 mM MgEGTA. Assuming 3 µM FH in serum, the molar ratio of rH:FH was ~20:1. Samples that received 10 mM EDTA were included as negative controls. C3 fragment deposition on neutrophils was determined as described in Section “Materials and Methods.” Results are representative of independent experiments done with neutrophils from different human volunteer donors that tested the entire rH fragment panel (indicated in each graph) and are graphed as mean and SD of duplicate observations, *n* = 2. The data were analyzed by one-way ANOVA with Dunnett’s multiple comparison test against MgEGTA alone. *p* < 0.05 (*) and *p* < 0.001 (***).

Recombinant fragments of FH (rH), spanning the length of the protein, were mixed with each cell type and serum under conditions that would specifically activate the AP. Fragments that can compete with FH for binding to the cell surface displace FH from the cell surface, leading to an increase in C3 fragments deposited on the cell. On both thrombin-activated platelets (Figure 1A) and neutrophils (Figure 1B), only rH19–20, composed solely of C-terminal domains 19–20, led to an increase in C3 fragments detected on the cell surface. RH19–20 increased C3 fragment deposition on platelets (Figure 1A) and neutrophils (Figure 1B) by ~3–4-fold and ~1.5–2-fold over cells incubated without rH fragments, respectively. Cumulatively, the data indicate the C-terminus is the most critical region of FH for protecting both isolated platelets and neutrophils from the AP. Moreover, the results indicate that rH19–20 can be used as an effective competitive inhibitor of FH function on these cell surfaces.

### Effects of FH on Neutrophils Are Complement-Dependent

**Figure 2:**
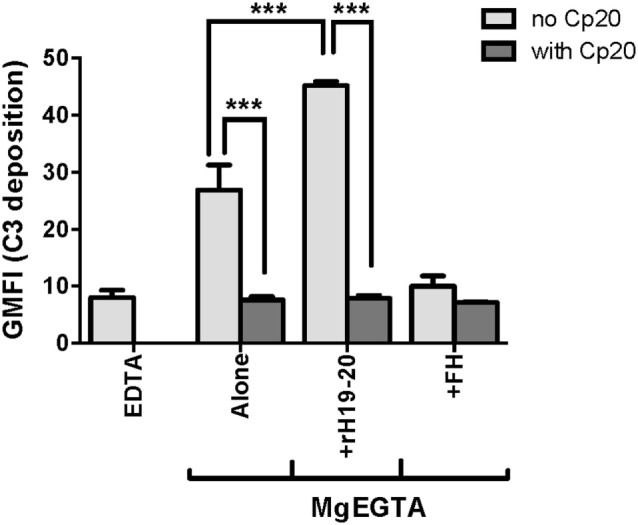
Effects of rH19–20 on C3 fragment deposition on neutrophils are complement-dependent. Neutrophils (2 × 10^6^/ml) were incubated without or with rH19–20 (20 µM) or Factor H (FH) (1.3 µM) ± Cp20 (50 µM) and 33% C8-depleted serum in the presence of 2.5 mM MgEGTA. Samples that received 10 mM EDTA were included as negative controls. C3 fragment deposition on neutrophils was determined as described in Section “Materials and Methods.” Results are representative of two independent experiments done with neutrophils from different human volunteer donors and are graphed as mean and SD of duplicate observations. The data were analyzed by one-way ANOVA with Tukey’s multiple comparison test. *p* < 0.001 (***).

Because FH has been reported to enhance neutrophil degranulation/activation by binding to complement receptor 3 (CR3; CD11b/CD18; Mac-1) (16, 37, 38), we determined whether rH19–20 or FH had any complement-independent functions in our *in vitro* neutrophil experiments. Cp20, a potent compstatin analog that inhibits all convertase-mediated complement activation (29), completely abrogated C3 fragment deposition in the absence or presence of rH19–20 (Figure 2). Furthermore, the addition of ~200 μg/ml FH reduced C3 fragment deposition to approximately the same level as the EDTA control and did not enhance C3 fragment deposition on neutrophils in the presence of Cp20. This indicates that in our experimental set-up, the effect of rH19–20 (and exogenous FH) is restricted to its ability to dysregulate AP activity through inhibition of FH cell surface interactions.

### Domains 19–20 Are Most Critical for Limiting TRAP-Mediated PGA Formation

**Figure 3:**
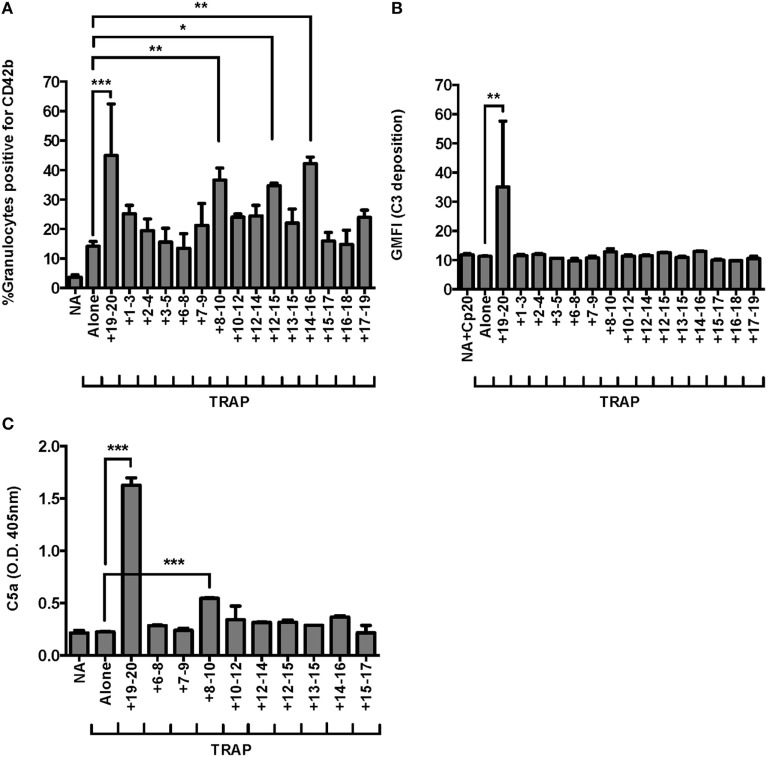
Domains 19–20 are critical for regulation of platelet/granulocyte aggregate (PGA) formation and alternative pathway activity in thrombin receptor-activating peptide (TRAP)-stimulated whole blood. 20 µl of lepirudin anticoagulated whole blood was incubated with TRAP (10 µM) ± rH fragments (20 µM), in a total 80 µl volume. Assuming 3 µM Factor H (FH) in each donors’ plasma and 60% plasma per blood sample, the molar ratio of rH:FH was ~44:1. Samples that did not receive TRAP [non-activated (NA)] and that were preincubated with Cp20 (NA + Cp20) were included in **(A–C)**. **(A)** PGA formation, **(B)** C3 fragment deposition, and **(C)** C5a generation were determined as described in Section “Materials and Methods.” **(A)** Graph is representative of at least three independent experiments (out of seven) in which rH8–10, rH12–15, and rH14–16 showed significant increases in PGA formation. **(B)** Graph is representative of seven independent experiments in which only rH19–20 showed significant increases in C3 fragment deposition in all seven experiments. **(C)** Graph is representative of three independent experiments in which rH8–10 and rH19–20 showed significant increases in C5a generation in all three experiments. The data were analyzed by one-way ANOVA with Dunnett’s multiple comparison test against TRAP alone. *p* < 0.05 (*), *p* < 0.01 (**), and *p* < 0.001 (***).

We previously demonstrated domains 19–20 were critical for FH regulation on granulocytes in TRAP-stimulated whole blood (25), however, it is unknown whether other regions of FH help anchor the protein to the granulocyte surface. FH contains C3b and polyanion binding domains in its central region, however, the location and number of these binding domains remains controversial (5, 26, 39, 40). We used our whole blood PGA assay to achieve very high competitive molar ratios of recombinant fragments to FH (~44:1, rH:FH), in order to detect potential weak effects of domains in the contested region of FH. Three fragments other than rH19–20 led to significant increases in TRAP-mediated PGA formation in at least three of seven experiments: rH8–10, rH12–15, and rH14–16. Figure 3A is representative of an experiment in which all three of these fragments significantly increased TRAP-mediated PGA formation. In all seven experiments, rH19–20 significantly increased PGA formation relative to TRAP alone and the increase in PGA formation was always greater than for any other fragment (Figure 3A), indicating the C-terminus is the most critical region of FH for regulating PGA formation in TRAP-stimulated human whole blood. Moreover, rH19–20 was the only fragment that increased C3 fragment deposition on granulocytes relative to TRAP alone (Figure 3B), which is consistent with its critical role in mediating regulation of the AP on both platelets and neutrophils (Figure 1). No other fragment had a statistically significant effect on C3 fragment deposition in any of seven experiments (Figure 3B). Because C5a is the key complement effector molecule for PGA formation (23–25), C5a levels correlate with PGA formation (25), and C5a detection is more sensitive in our hands than detection of C3 fragment deposition, we measured C5a levels in PGA reaction supernatants in the presence of fragments containing domains spanning from 6 to 17 as a more sensitive way to determine whether the effects of the fragments on PGA formation were complement-dependent. RH19–20 led to the greatest increase in C5a levels, up to an ~7-fold increase over TRAP alone (Figure 3C). Of the three other fragments that led to increases in PGA formation, only rH8–10 led to significant increases in C5a generation in all three experiments, although the fold-increase in C5a over TRAP alone was significantly lower than for rH19–20 alone (~1.5-fold) (Figure 3C). Collectively, the results suggest that of the three fragments of interest (rH8–10, rH12–15, and rH14–16), rH8–10 is actually competing with FH, while the effects of the other fragments require further study to determine the real biological significance of their contribution and may depend on the level of circulating FH in each donor’s blood. Fragments rH6–8, rH7–9, rH12–14, rH13–15, and rH15–17, which also contain domains previously described to have weak C3b and/or polyanion binding regions (5, 26, 39, 40) had no effect on PGA formation, C3 fragment deposition, or C5a generation relative to TRAP alone (Figures 3A–C), reducing the possibility of non-specific effects of rH8–10, rH12–15, and rH14–16 due to their polyhistidine tags. Cumulatively, these data indicate the C-terminus is the most important region for targeting FH to the platelet/granulocyte interface, although weak interactions with domains between 8 and 16 may enhance FH cell-surface protection.

### C5a Is the Key Complement Effector Molecule for PGA Formation When FH Cell Surface Protection Is Dysregulated

**Figure 4:**
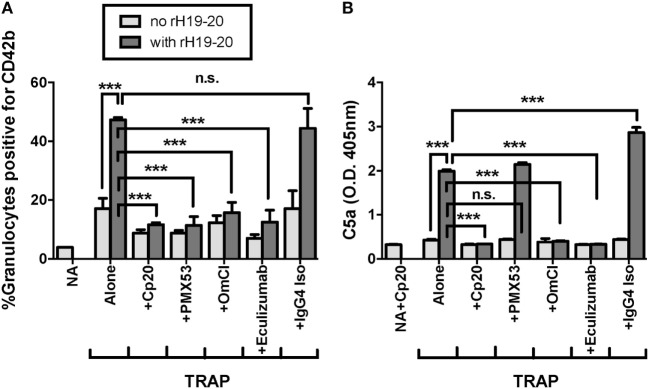
C5a is the key effector molecule for platelet/granulocyte aggregate (PGA) formation in the presence of dysregulated Factor H (FH) cell surface protection. 20 µl of lepirudin anticoagulated whole blood was incubated with thrombin receptor-activating peptide (TRAP) (10 µM) without or with rH19–20 (10 µM) ± anticomplement reagents (Cp20: 50 µM, PMX53: 16.7 µM, OmCI: 0.5 µM, and Eculizumab and IgG4 isotype control: 100 µg/ml), in a total 80 µl volume. Assuming 3 µM FH in each donors’ plasma and 60% plasma per blood sample, the molar ratio of rH:FH was ~22:1. A sample that did not receive TRAP [non-activated (NA)] or that was preincubated with Cp20 (NA + Cp20) was included for A and B, respectively. **(A)** PGA formation and **(B)** C5a generation were determined as described in Section “Materials and Methods.” Graphs are representative of **(A)** three and **(B)** two independent experiments done with blood from different human volunteer donors. Results are shown as mean and SD of duplicate observations. The data were analyzed by one-way ANOVA with Tukey’s multiple comparison test. *p* < 0.001 (***) and *p* > 0.05 (n.s.).

C5a is the key complement effector molecule influencing TRAP-mediated PGA formation under normal regulation (23–25). Additional complement-mediated mechanisms involved in the interaction between platelets and granulocytes include the ability of neutrophils to bind platelets through a CR3-C3(H_2_O) interaction (24), and enhanced platelet activation by MAC (41–43), both of which could account for some of the effect of complement on PGA formation. Because decreased FH cell surface protection increases the generation of all complement effector molecules, we sought to determine whether C5a remained the key complement effector molecule for enhancing PGA formation in the presence of rH19–20. Therefore, we tested the ability of PMX53, a C5a receptor 1 (C5aR1) antagonist previously shown to inhibit properdin-mediated increases in PGA formation (25), to inhibit rH19–20-mediated increases in PGA formation. Eculizumab and OmCI, two reagents that prevent C5 cleavage, were included to determine whether there were potential differences between inhibition of only C5a function (with PMX53) versus inhibition of both C5a and MAC formation. Eculizumab is a humanized recombinant IgG2/IgG4 anti-C5 monoclonal antibody, and OmCI is a tick salivary protein (32). Cp20 was included as a positive control for complete complement inhibition, and an IgG4 isotype control antibody was used as a control for Eculizumab. PMX53, Eculizumab, and OmCI, but not the IgG4 isotype control, completely abrogated the effects of rH19–20 on PGA formation, decreasing PGA formation to the level seen in samples receiving Cp20 (Figure 4A). The inhibitory reagents also completely abrogated rH19–20-mediated increases in C5a generation, with PMX53 being the only exception, as expected, since it inhibits C5a function rather than its generation (Figure 4B). The data indicate that C5a-C5aR1 interactions play a critical role in enhancing PGA formation in the presence of dysregulated FH cell surface protection.

### Effects of rH19–20 Are Primarily Mediated by Spontaneous AP Activity

**Figure 5:**
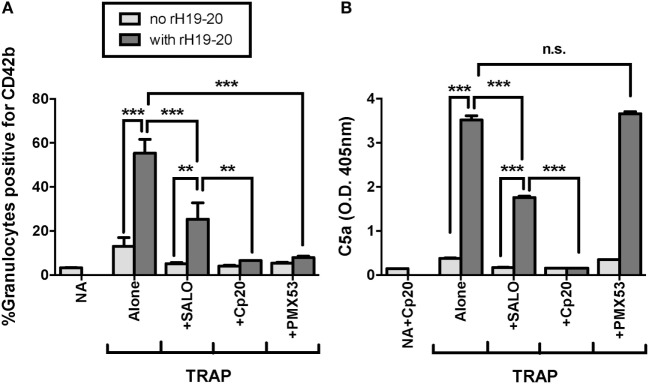
Effects of rH19–20 on platelet/granulocyte aggregate (PGA) formation are primarily mediated by C5a that is not generated by CP activity. 20 µl of lepirudin anticoagulated whole blood was incubated with thrombin receptor-activating peptide (TRAP) (10 µM) without or with rH19–20 (10 µM) ± anticomplement reagents (SALO: 2 µM, Cp20: 50 µM, PMX53: 16.7 µM), in a total 80 µl volume. Assuming 3 µM FH in each donors’ plasma and 60% plasma per blood sample, the molar ratio of rH:FH was ~22:1. A sample that did not receive TRAP [non-activated (NA)] or that was preincubated with Cp20 (NA + Cp20) was included for A and B, respectively. **(A)** PGA formation and **(B)** C5a generation were determined as described in Section “Materials and Methods.” Graphs are representative of three independent experiments done with blood from different human volunteer donors. Results are shown as mean and SD of duplicate observations. The data were analyzed by one-way ANOVA with Tukey’s multiple comparison test. *p* < 0.05 (*), *p* < 0.01 (**), *p* < 0.001 (***), and *p* > 0.05 (n.s.).

We previously showed that the effects of rH19–20 on PGA formation are completely abrogated by inhibition of properdin function or all AP activity (25). FH regulates AP activity, however, the AP can activate spontaneously or amplify complement activity initiated by the classical or lectin pathways. The classical pathway plays an important role in initiating complement activity when FH regulation is intact (25). To determine whether the classical pathway also plays a role in PGA formation when FH cell-surface protection is dysregulated, we tested the ability of SALO, a specific classical pathway inhibitor (31) at 2 µM (10–20-fold above the reported IC50), to limit rH19–20-mediated effects on PGA formation. Cp20 and PMX53 were included as controls for complete inhibition of PGA formation. Through 3 independent experiments, SALO inhibited the effects of rH19–20 on PGA formation by ~50%, whereas Cp20 and PMX53 completely abrogated the effects of rH19–20 (Figure 5A). These results were in agreement with the pattern of effects on C5a levels in reaction supernatants (Figure 5B). Our results combined with previous data (25) suggest that in the absence of FH cell surface protection, the effects of complement on PGA formation are primarily mediated by spontaneous AP activation, with a partial contribution from the classical pathway.

### AHUS-Related Mutations Have Differential Effects on Control of PGA Formation and Complement Activity in TRAP-Stimulated Whole Blood

**Figure 6:**
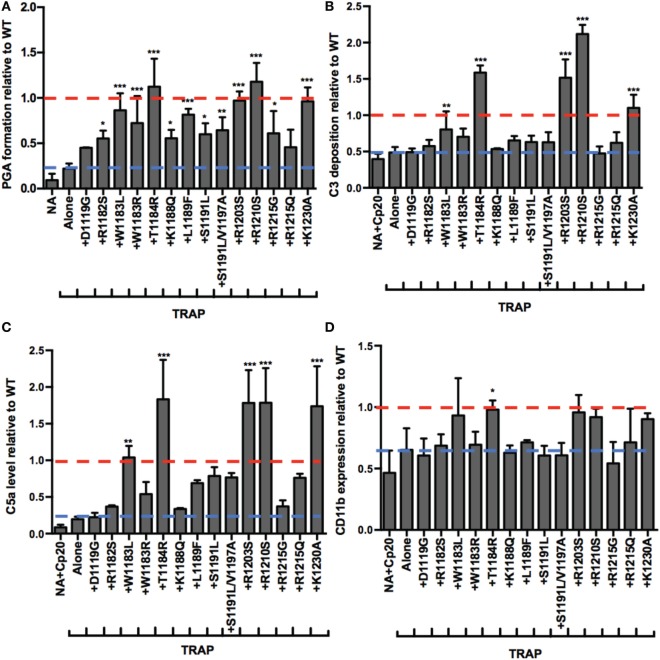
RH19–20 mutants have differential effects on platelet/granulocyte aggregate (PGA) formation and alternative pathway activity in thrombin receptor-activating peptide (TRAP)-stimulated whole blood. 20 µl of lepirudin anticoagulated whole blood was incubated with TRAP (10 µM) in the absence or presence of wild-type (WT) or mutant rH19–20 constructs (10 µM), in a total 80 µl volume. Assuming 3 µM Factor H (FH) in each donors’ plasma and 60% plasma per blood sample, the molar ratio of rH:FH was ~22:1. A sample that did not receive TRAP [non-activated (NA)] and that was preincubated with Cp20 (NA + Cp20) was included for A and B-D, respectively. **(A)** PGA formation, **(B)** C3 fragment deposition, **(C)** C5a generation, and **(D)** CD11b expression were determined as described in Section “Materials and Methods.” Each graph represents cumulative data from experiments carried out with different human volunteer blood donors (*n* = 3 for each mutant), and is graphed as the mean and SD for each mutant relative to WT (assigned a value of 1) for each independent experiment. Seven total experiments were conducted to test each mutant three times, therefore, the NA and TRAP alone groups represents the average value for all seven experiments. Red line: reference point for “+WT”; blue line: reference point for TRAP “Alone.” The four designer mutants that are not associated with disease (K1188Q, R1203S, R1210S, and K1230A) are grouped to the right of the graph. Data were analyzed by one-way ANOVA with Dunnett’s multiple comparison test against TRAP alone. *p* < 0.05 (*), *p* < 0.01 (**), and *p* < 0.001 (***).

Atypical hemolytic uremic syndrome is a prothrombotic disease associated with mutations mainly in FH domains 19–20 (19). The addition of rH19–20 to whole blood simulates pathophysiologic mechanisms involved in aHUS by competitively impairing FH cell surface protection, without affecting fluid phase regulation (8). Since rH19–20 leads to increases in TRAP-mediated PGA formation, this suggests that increased PGA formation may contribute to the thrombosis seen in patients with aHUS, therefore, we next assessed the effects of aHUS-related mutations in domains 19–20 on FH control of PGA formation. We utilized 14 rH19–20 constructs containing mutations that alter the affinity of the constructs for C3b and/or heparin (9). Ten mutations are associated with aHUS in humans (44), whereas the remaining 4 mutations (K1188Q, R1203S, R1210S, and K1230A) were previously designed to investigate the C3b and polyanion-binding sites of the FH C-terminus (9) and have not been associated with disease. When added to TRAP-stimulated whole blood, the average effects of the mutants on PGA formation (Figure 6A), C3 fragment deposition (Figure 6B), C5a generation (Figure 6C), and CD11b expression (Figure 6D) varied. The average level of PGA formation was higher (albeit with varying significance) in the presence of all mutants except D1119G and R1215Q compared to the average level of PGA formation seen with TRAP alone (Figure 6A). This indicates that the majority of mutants can at least partially compete with FH and induce PGA formation despite having altered affinities for C3b and heparin (when measured individually) (9). Five mutants (W1183L, T1184R, R1203S, R1210S, and K1230A) significantly increased C3 fragment deposition compared to TRAP alone (Figure 6B). Four of these 5 mutants (all but W1183L), led to average C3 fragment levels equal to or greater than the average level seen with WT (assigned a value of 1). The same five mutants that led to significant increases in C3 fragment deposition also significantly increased C5a levels compared to TRAP alone (Figure 6C). The average level of C5a in the supernatants of samples receiving these five mutants were greater than or equal to the average level seen with WT (assigned a value of 1).

**Figure 7:**
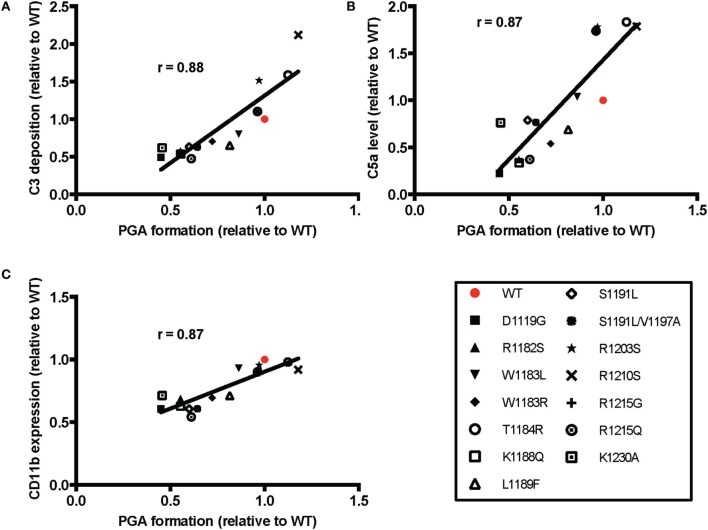
Effects of rH19–20 mutants on platelet/granulocyte aggregate (PGA) formation correlate with effects on C3 fragment deposition, C5a generation, and CD11b expression. The mean effect of each mutant on PGA formation relative to wild-type (WT) (red; assigned a value of 1) is graphed on the *X*-axis, and the mean effect of each mutant on **(A)** C3 fragment deposition, **(B)** C5a generation, and **(C)** CD11b expression relative to WT is graphed on the *Y*-axis. Data were analyzed by Pearson’s correlation test. The *r* value is displayed on each panel. *p* < 0.001 for all panels.

The average effects of the other mutants on C3 fragment deposition and C5a generation were more variable, with many failing to increase either parameter over the average level observed with TRAP alone. Specifically, mutants D1119G, R1182S, W1183R, K1188Q, S1191L, S1191L/V1197A, R1215G, and R1215Q resulted in no significant increases in average levels of C3 fragment deposition and C5a generation versus TRAP alone, indicating that these mutants (all aHUS related, except the designer mutant K1188Q) are affected in their ability to compete with normal FH-mediated protection. The average of effects of the mutants on CD11b expression were variable, but only T1184R led to significant increases in CD11b expression compared to TRAP alone (Figure 6D). This reflects the inconsistency in the effects of rH19–20 on CD11b expression, as observed previously (25). The average effects of the mutants on PGA formation did, however, correlate with the average effects of the mutants on CD11b expression (Figure 7C), as well as C3 fragment deposition (Figure 7A) and C5a generation (Figure 7B). Collectively, these data indicate that while the mutations do not universally impair the ability of the rH19–20 constructs to compete with FH in TRAP-stimulated whole blood, there are some mutations that may lead to enhanced PGA formation in patients with aHUS.

### Pattern of rH19–20 Mutant Impairment on Platelets and Neutrophils Is Similar to the Pattern Seen in Whole Blood

**Figure 8:**
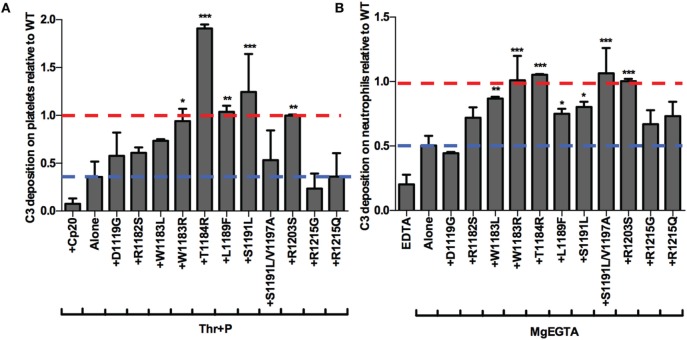
rH19–20 mutants have differential effects on control of the alternative pathway on platelets and neutrophils. **(A)** Thrombin-activated platelets preincubated with 1 µg/ml unfractionated properdin (Thr + P; 1 × 10^7^/ml) were incubated without or with wild-type (WT) or mutant rH19–20 constructs (25 µM) in 60% properdin-depleted serum in the presence of 5 mM MgEGTA. Assuming 3 µM Factor H (FH) in serum, the molar ratio of rH:FH was ~14:1. A sample that received Cp20 (50 µM) was also included. **(B)** Neutrophils (2 × 10^6^/ml) were incubated without or with WT or mutant rH19–20 constructs (20 µM) in 33% C8-depleted serum in the presence of 2.5 mM MgEGTA. Assuming 3 µM FH in serum, the molar ratio of rH:FH was ~20:1. A sample that received 10 mM EDTA was also included. C3 fragment deposition on **(A)** platelets and **(B)** neutrophils was determined as described in Section “Materials and Methods.” Each graph represents cumulative data from experiments carried out with different human volunteer blood donors (*n* = 2 for each mutant), and is graphed as the mean and SD for each mutant relative to WT (assigned a value of 1) for each independent experiment. Four total experiments were needed to test each mutant twice, therefore, the EDTA and MgEGTA alone groups represent the average for all four experiments. Red line: reference point for “+WT”; blue line: reference point for TRAP “Alone.” R1203S is a designer mutant not associated with disease. Data were analyzed by one-way ANOVA with Dunnett’s multiple comparison test against **(A)** Thr + P or **(B)** MgEGTA alone. *p* < 0.05 (*), *p* < 0.01 (**), and *p* < 0.001 (***).

Finally, we aimed to determine whether the mutants were preferentially impaired on either the surface of platelets or neutrophils. To that end, we examined the effects of 11 of the mutants on control of the AP on isolated platelets and neutrophils. Five mutants significantly increased C3 fragment deposition compared to thrombin-activated platelets with properdin alone (Figure 8A) and seven mutants significantly increased C3 fragment deposition compared to neutrophils incubated with MgEGTA alone (Figure 8B). T1184R and R1203S significantly increased C3 fragment deposition on both cells to a level that was approximately equal to or greater than the average level seen with WT (assigned a value of 1; Figures 8A,B), consistent with their statistically significant effects on PGA formation and AP activity in TRAP-stimulated whole blood (Figures 6A–C). Interestingly, W1183R significantly increased C3 fragment deposition on both cell types to approximately the same level as WT (Figures 8A,B), despite having no statistically significant effects on C3 fragment deposition or C5a generation in TRAP-stimulated whole blood (Figures 6B,C). L1189F and S1191L also had significant effects on C3 fragment deposition on both platelets (Figure 8A) and neutrophils (Figure 8B), while W1183L and S1191L/V1197A significantly increased C3 fragment deposition on neutrophils (Figure 8B). Mutants D1119G, R1182S, R1215G, and R1215Q were consistently affected in their ability to increase C3 deposition on both cell types, indicating that these aHUS-related mutants are impaired in their ability to effectively compete with FH for protection of these cell surfaces.

**Figure 9:**
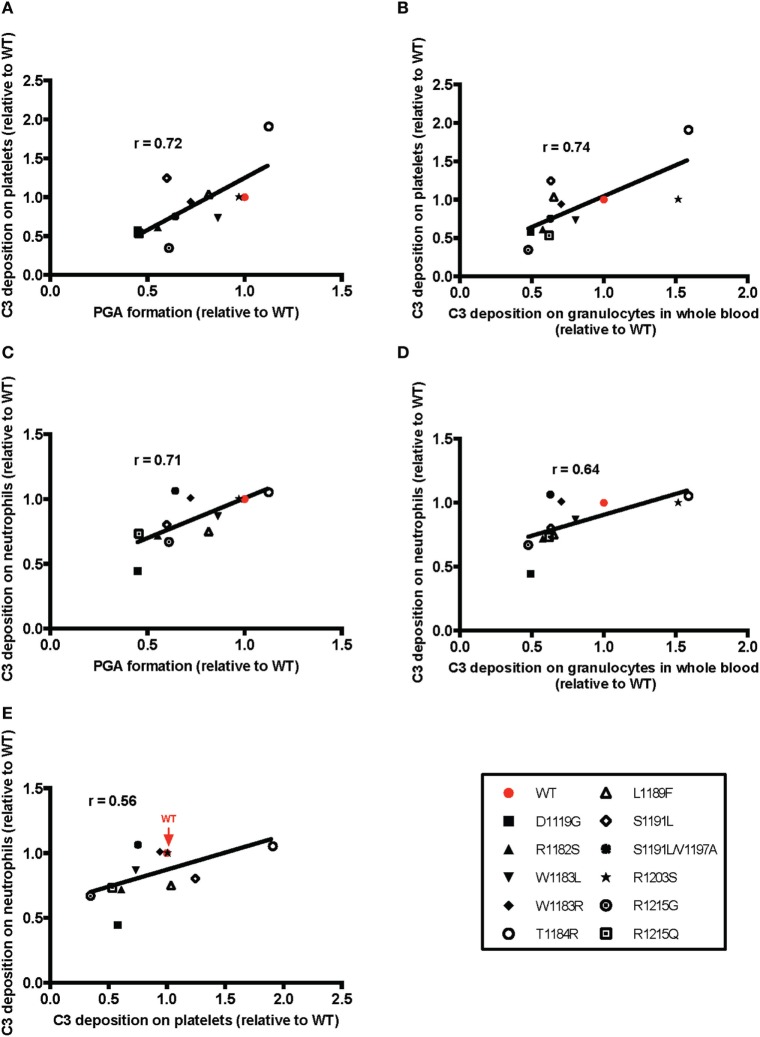
Correlation of the effects of mutants on platelet/granulocyte aggregate (PGA) formation and alternative pathway activity in thrombin receptor-activating peptide (TRAP)-stimulated whole blood with the effects on control of alternative pathway activity on isolated platelets and neutrophils. The mean effect of each mutant on **(A,C)** PGA formation or **(B,D)** C3 fragment deposition on granulocytes in TRAP-stimulated whole blood relative to wild-type (WT) (red; assigned a value of 1) is graphed on the *X*-axis, and the mean effect of each mutant on C3 fragment deposition on isolated **(A,B)** platelets and **(C,D)** neutrophils relative to WT is graphed on the *Y*-axis. **(E)** Graph represents mean effects of mutants on C3 fragment deposition relative to WT on platelets and neutrophils on *X* and *Y* axes, respectively. Data were analyzed by Pearson’s correlation test. The *r* value is displayed on each panel. **(A–D)** *p* < 0.05 and **(E)** n.s.

Overall, the pattern of the effects of the mutants was very similar to the pattern of their effects on PGA formation. The mutants that were least (T1184R, R1203S) or most (D1119G, R1182S, R1215G, R1215Q) impaired relative to WT in their effects on PGA formation, C3 fragment deposition, C5a generation, and CD11b expression (Figure 6), remained the least or most impaired in C3 fragment deposition relative to WT on the surface of both cell types (Figure 8). The average effects of the mutants on PGA formation and C3 fragment deposition in the whole blood PGA assay correlated with average effects of the mutants on C3 fragment deposition on isolated platelets (Figures 9A,C) and neutrophils (Figures 9B,D). There was not a significant correlation between the average effects of the mutants on C3 fragment deposition on isolated platelets and neutrophils (Figure 9E), suggesting that the mutants may recognize slightly different ligands on each cell surface. Altogether, results from Figures 6–9 indicate that aHUS-related mutations have differential effects on FH-mediated control of the AP on platelets and on neutrophils, which reflect their ability to control PGA formation. Therefore, FH is key for regulating PGA formation and AP activity on platelets and neutrophils and some aHUS-related mutations, including but not limited to D1119G, R1182S, R1215G, and R1215Q, may lead to an increased propensity to form PGAs that increase thrombosis in patients with the disease.

## Discussion

Atypical hemolytic uremic syndrome is a debilitating disease characterized by a triad of symptoms, including renal failure, hemolytic anemia, and excessive thrombosis leading to thrombocytopenia (19). Impaired FH cell surface protection is not only associated with aHUS, but also with increased PGA formation (25), a phenomenon that can cause pathologic thromboinflammation when in excess (20–22). Despite associations between decreased FH cell surface protection and aHUS, as well as increased PGA formation, it is unknown whether patients suffering from aHUS have increased levels of circulating PGAs in their blood. Here, we have elaborated on previous work that elucidated a key role for FH in controlling PGA formation (25), establishing the C-terminus as the most critical region of FH for controlling AP activity under thromboinflammatory conditions and identifying differential effects of aHUS-related mutations on PGA formation. Our data expand the current perception of regulatory mechanisms controlling PGA formation and has important implications for understanding the pathophysiologic mechanisms involved in aHUS.

*In vitro* mapping studies demonstrated that FH C-terminal domains are the most critical part of the protein for anchoring FH to activated platelets and neutrophils in order to limit C3 fragment deposition (Figures 1A,B). Our results were in agreement with previous mapping studies that also showed domains 19–20 were critical for binding to platelet and neutrophil surfaces (10, 11, 16). These reports, however, utilized isolated cells in the absence of a source of active complement and identified integrin receptors (GPIIb/IIIa; CR3) as key FH ligands. The surfaces of both activated platelets and neutrophils serve as a platform for the activation of the AP (12, 35), thus these studies neglected the role of C3b (a primary ligand for FH) deposited on cells under thromboinflammatory conditions in which the AP is activating. We confirmed a critical role of the C-terminus of FH in allowing FH to carry out its regulatory functions on the cell surface during complement activation (Figures 1 and 2). While domain 7 was shown to bind to both isolated platelets and neutrophils (10, 16), we detected no role for this domain on either cell type in the presence of active complement (Figure 1), suggesting that dual recognition of C3b and polyanions by the C-terminus is more critical than domain 7-mediated polyanion binding alone under the conditions used in this study. We did not use, however, a fragment containing domain 7 at the C-terminus as is present in FHL-1, a naturally truncated form of FH composed of domains 1–7 (2). C-terminal positioning of domain 7 may be relevant for effective interactions with the cell surface, thus we cannot exclude the possibility that FHL-1 may have a role in regulating PGA formation.

RH19–20 also had the most potent effects on PGA formation, C3 fragment deposition, and C5a generation in our *ex vivo* whole blood assay (Figure 3). Higher theoretical rH:FH ratios (~44:1, assuming a concentration of 3 µM FH in each donors’ plasma and 60% plasma per blood sample) in the whole blood assay allowed detection of weak but significant effects of fragments rH8–10, 12–15, and 14–16 on PGA formation in about half of our experiments, however, no effects were observed on C3 fragment deposition (Figures 3A,B). Furthermore, only rH8–10 consistently had effects on C5a generation (Figure 3C). Pangburn et al. previously identified a polyanion binding site within domains 11–15 (39) and demonstrated the importance of this region in limiting complement on self but not activating surfaces (6), thus the reasons for the lack of a clear complement-dependent effect on PGA formation by rH12–15 and rH14–16 remain a conundrum. The lack of effects on PGA formation by other fragments containing domains between 12 and 16 (e.g., rH12–14, rH13–15) likely rules out non-specific effects of rH12–15 and rH14–16 due to polyhistidine tags. RH12–15 and rH14–16 may contain unique binding sites due to the biochemical and structural properties of their individual domains that are not present on the other non-effective fragments. These binding sites may influence PGA formation *via* weak interactions with cell surface polyanions or other unknown ligands in TRAP-stimulated whole blood, which would need to be determined experimentally. The effects of rH8–10 may correspond to the C3b binding capability of domain 8 (5, 26), although it remains to be determined why rH6–8 would not show similar increases. The ability of rH8–10 to increase C5a generation without affecting C3 fragment deposition may be due to the greater sensitivity of the C5a detection assay in our hands. While further studies of the role of the middle region of FH are warranted, the lack of consistent and potent effects of fragments outside domains 19–20 on PGA formation and AP activity, despite very high competitive ratios of rH fragment to circulating FH in our system, indicate domains 19–20 are unequivocally the most important for targeting FH to the platelet/granulocyte interface and that other regions have a limited role.

Inhibition of C5 cleavage or blocking the interaction of C5a with C5aR1 completely abrogated rH19–20-mediated effects on PGA formation (Figure 4), indicating C5a remains the key effector molecule for PGA formation even when FH cell-surface regulation is impaired. While the CP plays a key role in initiating complement activity when FH regulation is intact (25), inhibition of the CP had only an intermediate effect in the presence of dyresgulated FH cell surface protection compared to inhibiting all complement activity or C5a-C5aR1 interactions (Figure 5). SALO reduced the effects of rH19–20 on PGA formation and C5a generation by about 50% (Figure 5), as opposed to inhibiting properdin or all AP activity, which completely abrogate the effects of rH19–20 on PGA formation and C3 fragment deposition (25). Together, results from Figures 4 and 5 and our previous study (25) suggest that in the absence of FH cell-surface protection in TRAP-stimulated whole blood, the CP plays a limited role in initiating complement activity and properdin-enhanced AP activity that generates C5a primarily accounts for the effects of complement on PGA formation. Our findings support targeted inhibition of the AP or C5a as a potential therapeutic option for patients with aHUS, and suggest that some of the benefits of inhibiting C5 in patients with, or in a mouse model of, aHUS may be a result of reduced platelet/granulocyte interactions (45, 46).

**Figure 10:**
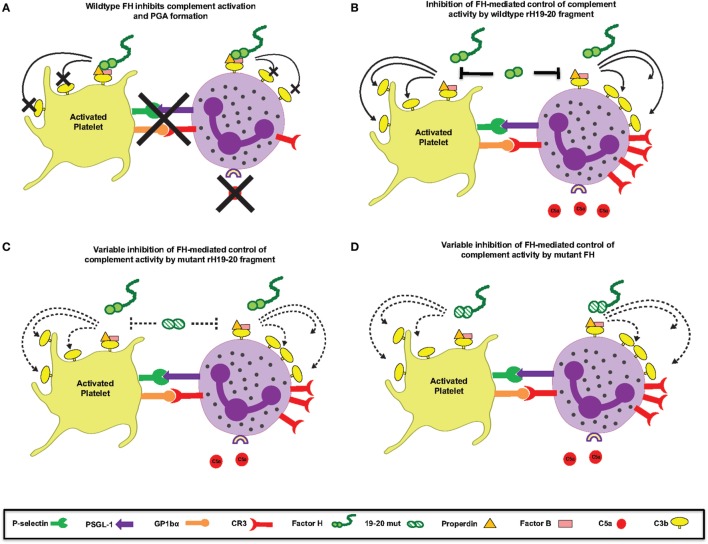
Schematic for Factor H (FH)-mediated control of complement at the platelet/granulocyte interface. **(A)** Wild-type (WT) FH negatively regulates complement activation and platelet/granulocyte aggregate (PGA) formation. **(B)** The rH19–20 fragment that is used in the *in vitro* assays competitively inhibits the ability of full-length FH to regulate complement activation, resulting in increased C5a generation, CR3 expression, and thus increased PGA formation. **(C)** The rH19–20 mutants used in the assays have variable ability to inhibit full-length FH-mediated complement regulation. Compared to WT rH19–20, the mutants may bind poorly to cells and have less impact on full-length FH-mediated protection from complement, resulting in reduced complement activation and PGA formation. If the rH19–20 mutant is not affected in his ability to bind, it will effectively compete with full-length FH, similar to WT rH19–20 (depicted in B), leading to increased complement activation and PGA formation. **(D)** Atypical hemolytic uremic syndrome-associated mutations in the C-terminus of full-length FH may have variable ability to bind to cells and will thus result in similar or increased PLA formation as compared to WT FH. Dotted lines represent variable effect on complement activation on cell surfaces or variable ability to bind/compete.

Mutations in domains 19–20 that are associated with aHUS have variable effects on binding to C3b and heparin when measured individually (9), meaning mutations could have variable effects on FH binding at the platelet/granulocyte interface. When evaluated in TRAP-stimulated whole blood, the 14 rH19–20 mutant constructs had differential effects on PGA formation, C3 fragment deposition, C5a generation, and CD11b expression (Figures 6 and 7). The pattern of effects of each mutant on PGA formation and C3 fragment deposition in TRAP-stimulated whole blood was similar to the pattern of effects on C3 fragment deposition on platelets and neutrophils *in vitro* (Figures 8 and 9). The moderate lack of correlation between C3 fragment deposition on isolated platelets and isolated neutrophils (*r* = 0.56; Figure 9E) suggests a possibility of FH binding not only to similar ligands, but also to ligands that are specific to either cell. For instance, FH can bind to pentraxin 3, which is secreted from neutrophil secondary granules, but not found in platelets (47). Effects of the mutants on C3 fragment deposition on isolated platelets (Figures 9A,B) and on isolated neutrophils (Figures 9C,D) each correlated with effects of the mutants on PGA formation and C3 fragment deposition on the granulocyte population (which includes PGAs) in TRAP-stimulated whole blood. Collectively, this suggests that FH control of the AP on both platelets and neutrophils contributes to regulation of TRAP-mediated PGA formation. Figure 10 shows a model for how full-length FH (Figure 10A), WT and mutant rH19–20 that are used in the *ex vivo* assays (Figures 10B,C), and full-length FH with C-terminal mutations (Figure 10D) participate in PGA formation.

The mutations had similar effects on control of the AP on human erythrocytes in a study by Ferreira et al. (9), indicating that while the magnitude of impairment may differ between cell surfaces, the relative effects of each mutation remain the same. In addition, Hyvarinen et al. tested the effects of three mutants used in our study (W1183L, T1184R, and R1215Q) on control of AP on platelets, and found T1184R to be unimpaired in its ability to compete with FH on platelets, while W1183L and R1215Q were impaired (13). These data are in good agreement with our results. The mutations can thus be divided into three subcategories: “high impairment,” “intermediate impairment,” and “low impairment.” Mutants D1119G, R1182S, R1215G, and R1215Q represent the “high impairment” group, as they were the mutants most impaired relative to WT on each cell surface. The designer mutant K1188Q, while not tested on platelets and neutrophils due to limited supplies, was one of the most impaired on human erythrocytes (9) and in controlling PGA formation (Figure 6), thus it would also likely fall into this group. Mutants R1203S and T1184R represent the “low impairment group.” These mutants were unimpaired or performed even better than WT in each assay tested. Based on their effects on PGA formation (Figure 6) and on lysis of human erythrocytes (9), R1210S and K1230A also fall into the “low impairment” group. The remaining mutations are in the “intermediate impairment” group, because they were either (1) intermediately impaired in each assay or (2) their effects varied depending on the assay. For instance, S1191L/V1197A significantly increased PGA formation (Figure 6A) and C3 fragment deposition on neutrophils (Figure 8B), but failed to significantly increase C3 fragment deposition on platelets (Figure 8A), relative to samples that did not receive rH19–20.

Of note, three of the four mutants in the “low impairment” group were the designer mutants R1203S, R1210S, and K1230A that were originally created to probe C3b and polyanion binding sites (9). Considering these mutants have not been associated with disease, it is not unexpected that these mutations had little effect in our assay. While these mutations cause differences in individual affinities to C3b and heparin (9), it appears that in a complex thromboinflammatory environment they have little to know effect on global FH function. The remaining designer mutant, K1188Q, was more impaired than the other designer mutants in our whole blood assay (Figure 6). This may reflect the location of this particular residue in the C-terminus, which resides in the sialic acid binding site (18), as opposed to R1203, R1210, and K1230 that reside outside the critical sialic acid contact points.

The mere location of K1188 likely does not entirely explain its effect on FH function. T1184 is located in the same functional site as K1188 in the C-terminus, a flexible region within the sialic acid binding site that does not make any direct contacts with the ligand (18). However, unlike K1188Q, T1184R was minimally impaired relative to WT in our assays, but has been associated with aHUS in genetic studies (19). The minimal effects of T1184R on FH function in our assays suggests T1184R may not, by itself, be a causative mutation for the development of aHUS and may require other factors such as additional complement-related mutations or sensitivity to specific triggers to develop disease. Indeed, the rare genetic variant T1184R has only been reported in one sporadic case of aHUS while the variants D1119G and R1215G, which have shown to have highly impaired function, are reported to segregate with disease in families with high penetrance.

The crystal structures for rH19–20 engaged with C3d (17) and sialic acid (18) offer insights into the effects of the mutations on binding to these ligands, yet there is no consensus as to how the structural data relates to the level of impairment on the cell surface. As stated, T1184 and K1188 are both within a region of the sialic acid binding site that is flexible but makes no direct contacts with sialic acid (18), but these mutants have drastically different effects on PGA formation and AP activity (Figure 6). These mutations also have dramatically different effects on binding to C3b and heparin; K1188Q is impaired in binding to both, while T1184R binds with greater affinity than WT to each (9). Individual affinities to either C3b or heparin as a contributing factor for cell surface effects are also unlikely, as R1182S and R1203S have nearly identical affinities for C3b and heparin (9), but have completely different effects on PGA formation (Figure 6). Hyvarinen et al. showed that some mutations resulted in specific impairment in binding to sialic acid on cell surfaces (13), however, a universal mechanism for the effects of aHUS-related mutations on control of the AP on erythrocytes, platelets, and endothelial cells (cell types effected in aHUS) remains to be determined.

The effects that aHUS-related mutations have on binding to polyanions other than sialic acid and heparin remain poorly understood. Chondroitin-4-sulfate is the primary glycosaminoglycan (GAG) on both platelets and neutrophils (48), and platelets secrete high concentrations of chondroitin-4-sulfate into the microenvironment upon activation (23). FH binds to chondroitin-4-sulfate (37, 49) and Herbert et al. demonstrated that rH19–20 mutants S1191L and S1191L/V1197A were unimpaired in binding to the related chondroitin-6-sulfate, among other polyanions (50). This raises the possibility that FH C-terminal domains may utilize GAGs in conjunction with C3b to bind to platelets and neutrophils, and that mutations in the C-terminal domains have differential effects on the ability of the mutants to bind and protect each cell. This has important implications for complement control in the vasculature, as both platelets and neutrophils activate the AP on their surface to aid in the thromboinflammatory response (12, 35). Depending on the mutation, AP activation could preferentially occur on platelets versus neutrophils, or vice versa, and patients could benefit from platelet- or neutrophil-targeted therapeutics. This also raises the possibility that mutations could be inserted into domains 19–20 of the developing therapeutic, mini-FH (51, 52), in order to target it to specific cell types. For instance, T1184R binds very strongly to platelets and neutrophils (Figure 8), thus a T1184R-mini-FH construct may enhance regulation of AP activity specifically on platelets and neutrophils at thromboinflammatory locations, thus limiting its retention at other locations in the body. More detailed studies into the effects of each mutation on cell- and tissue-specific binding are necessary to evaluate this possibility.

Our data suggest that in patients suffering from aHUS who have mutations in the “high impairment” group, increased PGA formation could contribute to excessive thrombosis. These patients may benefit from anti-inflammatory medications, including anti-complement therapeutics, to decrease thromboinflammatory effects of activated neutrophils in the vasculature, although clinical studies should be conducted to determine the extent of PGA formation in these patients. Because the tubular epithelium has a limited capacity to restrain complement activation (53), mutations in the “low impairment” are likely still impaired in their ability to protect the renal tubular epithelium. It is also possible that some aHUS-related mutations prevent FH oligomerization on the tubular epithelium (28). Based on the critical role of FH in protecting the tubular epithelium, FH oligomerization could be an essential step in providing effective control of the AP. Effects of aHUS-related mutations on FH oligomerization are unknown.

In conclusion, our data indicate FH C-terminal domains 19 and 20 are critical for controlling PGA formation and AP activation on platelets and neutrophils and that mutations in the C-terminal domains have differential effects on PGA formation. Our data have important implications for understanding the pathophysiologic mechanisms involved in aHUS and diseases characterized by increased PGA formation, and suggests that future studies should consider the contribution of platelet/granulocyte interactions in the manifestation of aHUS.

## Ethics Statement

This study was carried out in accordance with the recommendations of the Institutional Review Board from the University of Toledo College of Medicine and Life Sciences, with written informed consent from all subjects. All subjects gave written informed consent in accordance with the Declaration of Helsinki. The protocol was approved by the University of Toledo College of Medicine and Life Sciences Institutional Review Board.

## Author Contributions

Experiments were designed by AB, GS, and VF and conducted by AB and GS. Key reagents were generated and provided by AH, CC, DK, DR, and JL. The manuscript was written by AB and VF. All authors critically reviewed the manuscript and made key contributions to the analysis and interpretation of results.

## Conflict of Interest Statement

JL is the founder of Amyndas Pharmaceuticals, which is developing complement inhibitors (including third-generation compstatin analogs such as AMY-101). JL and DR are inventors of patents or patent applications that describe the use of complement inhibitors for therapeutic purposes, some of which are developed by Amyndas Pharmaceuticals. JL is also the inventor of the compstatin technology licensed to Apellis Pharmaceuticals [i.e., 4(1MeW)7W/POT-4/APL-1 and PEGylated derivatives such as APL-2]. The remaining authors have no conflict of interest to declare.
